# Functional roles of sphingolipids in immunity and their implication in disease

**DOI:** 10.1038/s12276-023-01018-9

**Published:** 2023-06-01

**Authors:** Mingyu Lee, Suh Yeon Lee, Yoe-Sik Bae

**Affiliations:** 1 Department of Health Sciences and Technology, SAIHST, Sungkyunkwan University, Seoul, 06355 Republic of Korea; 2 Department of Biological Sciences, Sungkyunkwan University, Suwon, 16419 Republic of Korea

**Keywords:** Lipid signalling, Mechanisms of disease, Diagnostic markers

## Abstract

Sphingolipids, which are components of cellular membranes and organ tissues, can be synthesized or degraded to modulate cellular responses according to environmental cues, and the balance among the different sphingolipids is important for directing immune responses, regardless of whether they originate, as intra- or extracellular immune events. Recent progress in multiomics-based analyses and methodological approaches has revealed that human health and diseases are closely related to the homeostasis of sphingolipid metabolism, and disease-specific alterations in sphingolipids and related enzymes can be prognostic markers of human disease progression. Accumulating human clinical data from genome-wide association studies and preclinical data from disease models provide support for the notion that sphingolipids are the missing pieces that supplement our understanding of immune responses and diseases in which the functions of the involved proteins and nucleotides have been established. In this review, we analyze sphingolipid-related enzymes and reported human diseases to understand the important roles of sphingolipid metabolism. We discuss the defects and alterations in sphingolipid metabolism in human disease, along with functional roles in immune cells. We also introduce several methodological approaches and provide summaries of research on sphingolipid modulators in this review that should be helpful in studying the roles of sphingolipids in preclinical studies for the investigation of experimental and molecular medicines.

## Immunology: Membrane lipids in immunity and disease

Advances in laboratory techniques and genetic databases are revealing the vital roles played by a large and complex family of lipids in immunity and disease. Sphingolipids, found in cell membranes, respond to environnmental cues to control cell signaling and metabolism. Initially named after the Sphinx because of their enigmatic nature, their complexity made detection difficult. Yoe-Sik Bae and co-workers at Sungkyunkwan University in Seoul, South Korea, have reviewed the discoveries made through methods such as mass spectrometry and integrated dataset analyses (‘multi-omics’). For example, sphingolipid levels in the blood are affected by gut bacteria, cellular levels can determine whether cells live or die, and changes in sphingolipid populations have been linked to several diseases. Furthermore, analyzing the sphingolipid content in blood could aid diagnosis, because damaged organs express unique patterns of sphingolipid products.

## Introduction

The immune system is a complex network of white blood cells, organs, and secreted molecules that recognize allies/intruders, selectively excluding potential sources of damage and thereby protecting the host organism from dysfunctions known as diseases. The radar and tactical teams of the immune system need to act immediately and swiftly while fulfilling long-term needs with accurate preparedness against urgent or upcoming threats^1^. The innate and adaptive immune systems take part in these different tasks and provide fast and unspecific or accurate and tailored immune responses, respectively, gradually complementing mutual inadequacies with characteristics such as trained immunity and immunological memory^1,2^. During this process, each cell within inflamed tissues and recruited immune cells mark their state and communicate with signals for proper immune responses, expressed as surface checkpoint marker proteins and/or secreted cytokine profiles, while controlling their own signaling cascades according to the immune context^1–3^.

The plasma membrane and organelles (in the case of membrane-bound organelles in eukaryotic cells) are dense networks of lipids and proteins encasing a cell or within a cell, respectively, that insulate the inner spaces from the outer spaces for proper function. Lipids provide and maintain the lipid bilayer structure that separates specific areas and provides the appropriate circumstances for the cells to function due to their amphipathic nature stemming from the polarized structure of lipids with hydrophilic heads and hydrophobic tails and incorporated proteins^4–6^. There are three major lipids that comprise the structure of lipid bilayers. The most common lipid is glycerophospholipids (GPLs), although the composition of each lipid bilayer differs depending on cell type and distribution in tissues. GPLs are the main structural components of plasma membranes and are composed of diacylglycerol-linked hydrophobic fatty acid chains, which are usually unsaturated and centered in the membrane, and diverse hydrophilic head groups, which constitute the inner and outer surfaces of the membrane. Not restricted to the structural frame, GPLs can also act as storage anchors for signaling molecules and their precursors to modulate the functions of neighboring proteins such as receptors and transporters^5^. Sterols, a subgroup of steroids composed of fused rings with hydrocarbon tails and hydroxyl groups, which mainly exist as cholesterol in mammals, are incorporated into stacked lipid bilayers to sustain/stabilize the chemistry of the lipid bilayer and can be converted into biological molecules such as hormones^5,6^. Finally, sphingolipids, composed of usually long and saturated *N*-acyl hydrocarbon chains, a sphingoid base, and a head group, can recognize environmental cues and be degraded/synthesized according to cellular needs, thus playing crucial roles in signal transduction and modulation of cell metabolism^4,5^. Collectively, the compositional diversity of membrane lipids can act as complex codes or conditional cues of the cells, and these different patterns can decide/modulate biological functions through the interactions of lipids and proteins, such as internal/external signaling molecules and receptors. In this review, we shed light on recent progress in understanding the roles of membrane-derived lipids, especially sphingolipids, in the functions of immune cells and related diseases with clinically used/tried medicines.

## Sphingolipids as “protean” signaling modulators

**Fig. 1:**
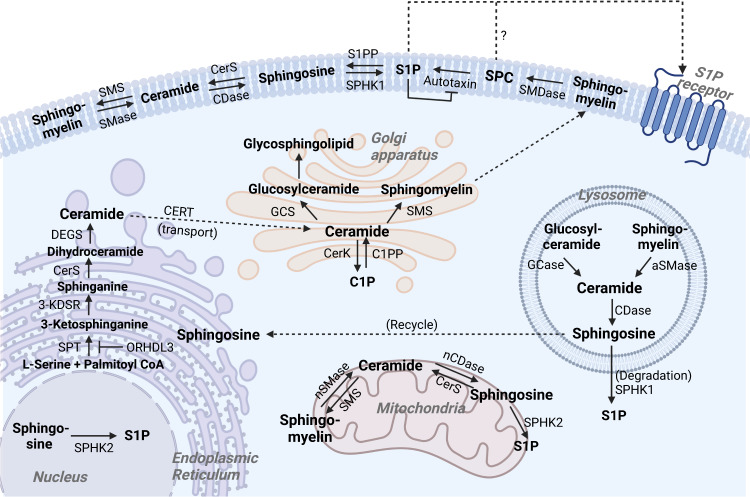
Synthesis and degradation of sphingolipids in cellular regions. Sphingolipids, as components of cellular and subcellular membranes, can be synthesized by serial enzymes (de novo pathway) and can also be degraded from sphingomyelin and ceramide to smaller sphingolipids (salvage pathway) as signaling molecules according to the environmental context.

Considering the roles of sphingolipids as secondary messengers in intra- and extracellular spaces, monitoring the synthesis/breakdown of sphingolipids and sphingolipid-related enzyme activities are important clues for understanding signal transduction and the states of resting/responding cells^4,7^ (Fig. 1 and Table 1). Sphingomyelin, ceramides, and other lipids agglomerate to form the asymmetric but balanced lipid rafts across the cellular membrane^4^. Ceramides are highly concentrated and major sphingolipids in the plasma membrane and can be generated by de novo synthesis from palmitoyl-CoA and L-serine by the sequential reactions of serine palmitoyltransferase (SPT), ceramide synthase (CerS), and dihydroceramide desaturases at the cytosolic leaflet of the endoplasmic reticulum (ER). When transferred to the *trans*-Golgi apparatus, lysosome, and plasma membrane from the ER, ceramides can be converted to sphingomyelins by sphingomyelin synthase (encoded by the *SGMS* gene), and these sphingomyelins gradually increase in concentration to compose most of the sphingolipids of the plasma membrane, which respond to environmental cues as precursors of secondary signaling messengers^8^. Conversely, ceramides can be restored by the hydrolysis of sphingomyelins by different sphingomyelinases (SMases) in different locations^4,7,8^. Although ceramides themselves can act as bioactive signaling cues in a variety of physiological responses, they can be degraded into sphingosine and then transformed into sphingosine-1-phosphate (S1P) by the action of ceramidase and sphingosine kinases (SphK), respectively, or phosphorylated by ceramide kinase (CERK) to generate ceramide-1-phosphate (C1P)^9–11^. In a negative feedback loop, C1P can inhibit the activity of SMase and SPT, blocking the excessive accumulation of ceramides^11–13^. Conversely, ceramides can be recovered from C1P by C1P phosphatase and S1P and sphingosine by S1P phosphatase and CerS, referred to as the salvage pathway, diminishing the excessive production of their metabolites^4,11^. In an SMase-independent manner, sphingomyelin can be degraded into sphingosylphosphorylcholine (SPC) and then further transformed into S1P by the actions of sphingomyelin deacylase and autotaxin (ATX), the latter of which is an ectonucleotide pyrophosphatase (phosphodiesterase with lysophospholipase D activity), originally identified as a tumor-regulatory factor for survival and proliferation^14,15^. One interesting aspect of these processes is that the modulation of sphingolipid metabolism is not exclusively performed by the host. Recently, several studies have revealed and discussed that commensal or pathogenic microbiomes, especially the commensal Bacteroides, provide or hijack sphingolipids as building blocks and utilize the host machinery, thereby affecting the host’s sphingolipid content and immune responses^16,17^. Homeostasis among these sphingolipids, regardless of whether they originate from food, the host, or the microbiome, is important because transformed and selectively biased compositions of sphingolipids can decide the fate of cells, such as maintenance (death, survival, and proliferation), renewal, senescence, differentiation, migration/retention, immune responses, and cell metabolism, and these cells collectively make up tissues, organs, and organ systems and ultimately determine the life of the organism^16–18^.

**Table 1:** Molecules targeting sphingolipid metabolism and related human diseases.

Target enzyme and protein	Gene symbol (NCBI gene ID)		Target function	Modulator(s)	Related diseases (human case studies)
	*Homo sapiens*	*Mus musculus*			
Serine palmitoyltransferase (SPT)			Convert serine + palmitoyl coenzyme A to 3-keto-dihydrosphinganine (de novo synthesis)		
SPT, long-chain base subunit 1 (SPTLC1)	SPTLC1 (10558)	Sptlc1 (268656)	Large subunit of SPT, forms the catalytic core with SPTLC2/3.	**Enhancer**: Fenretinide; **Inhibitors**: Myriocin (ISP-1), Sphingofungin B, WXP-003, SPT-IN-1, L-Cycloserine, L-Penicillamine, Imidazopyridine, Pyrazolopyridine. Lipoxamycin (G-26146).	**Sensory neuropathy and motor neuron disease**. Neurodegenerative disease/Alzheimer’s disease. Clear cell renal cell carcinoma. Macular telangiectasia type 2. Psoriasis. Juvenile amyotrophic lateral sclerosis.
SPT, long-chain base subunit 2 (SPTLC2)	SPTLC2 (9517)	Sptlc1 (20773)	Component of the large subunit of SPT as SPTLC2/3, forms the catalytic core.		**Sensory neuropathy and motor neuron disease**. Macular telangiectasia type 2. Congenital insensitivity to pain. Aspirin-exacerbated respiratory disease.
SPT, long-chain base subunit 3 (SPTLC3)	SPTLC3 (55304)	Stplc3 (228677)			Metabolic disease. Type 2 diabetes.
SPT, small subunit A	SPTSSA (171546)	Sptssa (104725)	Either SPTSSA or SPTSSB increases the efficiency of the catalytic reaction.		–
SPT, small subunit B	SPTSSB (165679)	Sptssb (66183)			–
Ormdl sphingolipid biosynthesis regulator (ORMDL) 3	ORMDL3 (94103)	Ormdl3 (66612)	Negative regulation of de novo ceramide biosynthesis, blocking SPT activity.	–	**Asthma**. **Atopic dermatitis**. **Inflammatory bowel disease** (IBD, Crohn’s disease, Ulcerative colitis). **Primary biliary cholangitis**. Chronic obstructive pulmonary disease (COPD). Bronchiolitis. Respiratory syncytial virus infection. Aspirin-exacerbated respiratory disease. Allergic rhinitis. Ankylosing spondylitis. Atherosclerosis.
3-Keto-dihydrosphinganine reductase (3-KDSR)	KDSR (2531)	Kdsr (70750)	Reduces 3-ketodihydrosphiganine to dihydrosphingosine.	–	Palmoplantar keratoderma. Thrombocytopenia. Anemia. Juvenile myelofibrosis. Erythrokeratodermia.
Ceramide synthase (CerS)			N-acetylate dihydrosphingosine to dihydroceramide. Each CerS prefers a different length of fatty acid chains for the sphingoid base		
CerS1	CERS1 (10715)	Cers1 (93898)	Attaches C18 fatty acyl CoA to sphingoid base.	**Inhibitors**: FTY720, P053, Fumonisin B1, Australifungin.	Head and neck squamous cell carcinomas. Age-related disease. Myoclonus epilepsy. Dementia.
CerS2	CERS2 (29956)	Cers2 (76893)	Located in the ER and membrane. Attaches C22-24 fatty acyl CoA to sphingoid base. Tumor metastasis suppressor.	**Inhibitors**: FTY720, Fumonisin B1, ST1058, ST1074, Bcl2L13, Australifungin.	**Bladder cancer**. **Breast cancer**. **Tumor metastasis**. Liver cancer. Ovarian cancer. Renal cancer. Rhegmatogenous retinal detachment. Multiple sclerosis (MS).
CerS3	CERS3 (204219)	Cers3 (545975)	Located in ER. Attaches C26-34 fatty acyl CoA to sphingoid base. Important for epidermis to create barrier.	**Inhibitors**: Fumonisin B1, Australifungin.	**Congenital ichthyosis**. Prader-Willi syndrome.
CerS4	CERS4 (79603)	Cers4 (67260)	Located in ER. Attaches C18-20 fatty acyl CoA to sphingoid base.	**Inhibitors**: Fumonisin B1, ST1058, ST1072, ST1074, Australifungin.	Endometrial cancer. Colon cancer.
CerS5	CERS5 (91012)	Cers5 (71949)	Located in ER. Attaches C14-16 fatty acyl CoA to sphingoid base.	**Inhibitor**: Fumonisin B1, Australifungin.	Endometrial cancer. Colon cancer.
CerS6	CERS6 (253782)	Cers6 (241447)	Located in ER. Attaches C14-16 fatty acyl CoA to sphingoid base.	**Inhibitors**: Fumonisin B1, Australifungin, ST1072.	**Tumor metastasis**. **Breast cancer**. Lung cancer. Ovarian cancer. Melanoma. Gastric cancer. Pancreatic cancer. Renal cancer. Leukemia. Multiple sclerosis. T2D.
Dihydroceramide desaturase (DEGS)			Dehydrogenate dihydroceramide to ceramide.		
DEGS1	DEGS1 (8560)	Degs1 (13244)	Ceramide de novo synthesis. Positive regulator of apoptosis. Located in mitochondria.	**Inhibitors**: Fenretinide (4-HPR); XM462, GT11 (C8-cyclopropenylceramide), Celecoxib, γ-Tocotrienol.	**Hypomyelinating leukodystrophy**. Intellectual disability. Epilepsy. Spastic quadriplegia. Microcephaly. Scoliosis.
DEGS2	DEGS2 (123099)	Degs2 (70059)	Ceramide de novo synthesis and sphinganine metabolic process. Located in the ER and membrane.		Schizophrenia. Sudden cardiac arrest.
Ceramide transfer protein (CERT)	CERT1 (10087)	Cert1 (68018)	Transfers ceramide from the ER to the Golgi in a nonvesicle manner for sphingomyelin synthesis. Located in the cytosol and mitochondria.	**Inhibitors**: E16A/(1 S,2 R)-HPCB-5, HPA-12.	**Goodpasture syndrome**. Alport syndrome. Intellectual disability. Alzheimer’s disease. Triple-negative breast cancer. T2D.
Ceramide kinase (CERK)	CERK (64781)	Cerk (223753)	Converts ceramide to ceramide-1-phosphate (C1P). Located in mitochondria.	**Inhibitor**: NVP-231	**Breast cancer**. Tumor metastasis. Tumor recurrence. Cancer-drug resistance. Inherited retinal dystrophy.
C1P Transfer protein (CPTP)	CPTP (80772)	Cptp (79554)	Transfers C1P and sphingomyelin from the *trans*-Golgi to the membrane. Located in the Golgi, endosomes, nucleus, and cytosol.	–	Pancreatic cancer. Tumor metastasis.
Glycosylceramidase (GCase)			Hydrolyzes glucosylceramide to glucose and ceramide.		
Glycosylceramidase beta (GBA) 1	GBA1 (2629)	Gba (14466)	Enables glucosyltransferase and hydrolase activity. Located in the ER, lysosome, *trans*-Golgi.	**Inhibitor**: Conduritol B epoxide.	**Parkinson’s disease**. **Gaucher disease**. **Dementia with Lewy bodies**. Multiple system atrophy. REM-sleep behavior disorder. Atopic dermatitis. Krabbe disease. Liver cancer. Tumor metastasis. Rheumatoid arthritis. Psoriasis vulgaris.
GBA2	GBA2 (57704)	Gba2 (230101)	Located in cytosol, extrinsic membrane component of the Golgi, ER. Nonlysosomal.	**Inhibitor**: ACT-519276 (Sinbaglustat).	Hereditary spastic paraplegia. Autosomal-recessive cerebellar ataxia. Parkinson’s disease. Charcot-Marie-Tooth disease. Senile dementia. Melanoma. Sjögren’s syndrome.
Glucosylceramide synthase (GCS)	UGCG (7357)	Ugcg (22234)	Located in the Golgi and membrane. Glucosyltransferase activity. Transfers glucose to ceramide.	**Inhibitors**: d-threo-P4, *N*-butyl-deoxynojirimycin, PDMP, Genz-123346, Genz-529468, T-036, T-690, CCG-203586.	**Breast cancer**. Tumor metastasis. Cancer-drug resistance. Thyroid cancer. Colorectal cancer. Bladder cancer. Endometriosis. Alzheimer’s disease. Type 1 Gaucher disease.
Four-phosphate adapter protein 2 (FAPP2)	PLEKHA8 (84725)	Plekha8 (231999)	Regulates vesicular trafficking and transfers to *trans*-Golgi network and membrane.	–	Hepatitis C virus. Liver cancer. Colon cancer. T-cell acute lymphoblastic leukemia.
Sphingomyelin synthase (SMS)			Converts ceramide to sphingomyelin		
SMS1	SGMS1 (259230)	Sgms1 (208449)	Localized in the Golgi membrane, majorly contributes to sphingomyelin synthesis.	**Inhibitors**: Malabaricone C, SAPA 1j (SMS1-IN-1), D609	Liver cancer. Endometriosis. Systemic lupus erythematosus. Alzheimer’s disease.
SMS2	SGMS2 (166929)	Sgms2 (74442)	Localized in the Golgi and plasma membrane, synthesizes sphingomyelin primarily at the cell membrane.	**Inhibitors**: Malabaricone C, D2/Dy105, SMS2-IN-1, 15w, SMS2-IN-Ly93.	Osteoporosis. Skeletal dysplasia. Breast cancer. Tumor metastasis. Chronic obstructive pulmonary disease (COPD).
SMSr	SAMD8 (142891)	Samd8 (67630)	Sphingomyelin synthase (SMS)-related protein. Localized in the cytosol and ER membrane.	–	–
Sphingomyelinase (SMase)			Hydrolyze sphingomyelin to form ceramide		
Acid SMase (Lysosomal/secreted)	SMPD1 (6609)	Smpd1 (20597)	Lysosomal: Located in the lysosomal region and has zinc-dependent phosphodiesterase activity that produces ceramide from sphingomyelin.	**Inhibitors**: Ambroxol, ARC39, Fluoxetine (Prozac), Desipramine (Norpramin), Functional Inhibitors of Acid sphingomyelinase (FIASMA).	**Niemann-Pick disease**. **Parkinson’s disease**. **Depressive disorder**. Sepsis. Atopic dermatitis. Allergy. Alzheimer’s disease. Alcohol dependence. Hepatitis C. Nonalcoholic fatty liver disease (NAFLD). Scleroderma. Cancer-drug resistance. Multiple myeloma. Large granular lymphocyte leukemia. Lung cancer. Colorectal cancer. Respiratory syncytial virus infection. COVID-19. Rheumatoid arthritis.
			Secreted: Located in the extracellular region.		
Neutral SMase 1	SMPD2 (6610)	Smpd2 (20598)	Located in the membrane. Mediates the secretion of ceramide.	**Inhibitor**: GW4869.	Atopic dermatitis. Large granular lymphocyte leukemia. Liver cancer.
Neutral SMase 2	SMPD3 (55512)	Smpd3 (58994)	Located in the Golgi, cytoplasm and plasma membrane. Mediates the exosomal secretion of ceramide.	**Inhibitors**: DPTIP, Cambinol, GW4869, Altenusin.	Melanoma. Tumor metastasis. Acute myeloid leukemia. Alcohol dependence. Depressive disorder. Endometriosis. Systemic lupus erythematosus.
Neutral SMase 3	SMPD4 (55627)	Smpd4 (77626)	Located in the ER, Golgi and nuclear envelope. Mediates stress-induced cellular response.	–	Breast cancer. Lung cancer. Colon cancer. Microcephaly. Congenital arthrogryposis. Colorectal cancer. Kidney cancer.
Mitochondria-associated neutral SMase	SMPD5 (392275)	Smpd5 (100503915)	Located in the ER membrane and mitochondrial membrane.	–	–
Neutral SMase activation associated factor	NSMAF (8439)	Nsmaf (18201)	Mediates TNF and other stress-induced cellular responses.	–	Tuberculosis.
Alkaline SMase	ENPP7 (339221)	Enpp7 (238011)	Expressed in the intestinal mucosal region and liver but not in other tissues. Modulates the intestinal lipid metabolic process, digests dietary sphingomyelin. Exists in membrane-bound or secreted form.	–	Colon cancer.
Ceramidase			Cleaves ceramide to form sphingosine.		
Acid ceramidase	ASAH1 (427)	Asah1 (11886)	Alternative splicing generates alpha and glycosylated beta subunit to form lysosomal enzyme. A heterodimer of two isoforms hydrolyzes ceramide into sphingosine.	**Inhibitors**: Cystatin SA, ARN14988, B-13, LCL-521.	**Faber disease**. **Myoclonic epilepsy**. **Muscular dystrophy**. **Prostate cancer**. **Breast cancer**. **Tumor metastasis**. **Melanoma**. **Radiotherapy failure**. Rectal cancer. Schizophrenia. Ovarian cancer. Head and neck cancer. Acute myeloid leukemia. Ependymoma. Large granular lymphocyte leukemia. Keloid. Atherosclerosis. Obesity.
Neutral ceramidase	ASAH2 (56624)	Asah2 (54447)	Located in the extracellular space, mitochondria, caveolae	**Inhibitors**: B-13, LCL-464.	–
Alkaline ceramidase 1	ACER1 (125981)	Acer1 (171168)	Highly expressed in the epidermis. Hydrolyzes very long-chain ceramides.	**Inhibitor**: D-erythro-MAPP.	–
Alkaline ceramidase 2	ACER2 (340484)	Acer2 (230379)	Located in the Golgi membrane. Hydrolyzes very long-chain ceramides.		Breast cancer.
Alkaline ceramidase 3	ACER3 (55331)	Acer3 (66190)	Located in the ER, Golgi membrane.		**Leukodystrophy**. Liver cancer. Acute myeloid leukemia. Glioma.
Sphingosine kinase (SphK)			ATP-dependently phosphorylates sphingosine to sphingosine-1-phosphate (S1P).		
SphK1	SPHK1 (8877)	Sphk1 (20698)	Located in the cytosol and plasma membrane. Involved in TNF- and NF-kB-mediated inflammatory responses.	**Inhibitors**: Safingol, SKI-I, SKI-II, PF-543, DMS (N,N-dimethylsphingosine).	**Tumor metastasis**. **Breast Cancer**. **Gastroesophageal carcinoma**. **Head and neck carcinoma/Oral squamous cell carcinoma**. **Liver cancer**. **Colorectal cancer**. **Prostate cancer**. Chemo-resistance. Tumor recurrence. Thyroid cancer. Colon cancer. Alzheimer’s disease. Autoimmune Thyroiditis. Huntington’s disease. Glioblastoma. Melanoma. Large granular lymphocyte leukemia. Acute myeloid leukemia. Immune checkpoint inhibitor-mediated antitumor efficacy. Salivary gland carcinoma. Astrocytoma. Portal vein tumor thrombus. Pancreatic cancer. Gastric cancer. Colitis-associated cancer. Bladder cancer. Nasopharyngeal carcinoma. Sacral chordoma. Uterine cervical cancer. Lung cancer. Tumor angiogenesis. Hepatitis B. Liver fibrosis. Pulmonary edema. Idiopathic pulmonary fibrosis. Spondyloarthritis. Diabetic nephropathy. T2D. Insulin resistance. Niemann-Pick disease. Sickle cell disease.
SphK2	SPHK2 (56848)	Sphk2 (56632)	Located in the nucleus. Several tumor cells highly express SphK2.	**Inhibitors**: ABC294640, SKI-II, K-145. MP-A08, ROME (R-FTY720 methyl ether).	**Tumor metastasis**. Liver cancer. Gastric cancer. Glioblastoma. Lung cancer. Alzheimer’s disease. Chemo-resistance. Neuroblastoma. Breast cancer. Colorectal cancer. Large granular lymphocyte leukemia. Spondyloarthritis. Osteoarthritis. Rheumatoid arthritis. Hypertrophic scar. Alcoholic cirrhosis.
Spinster (SPNS) 2	SPNS2 (124976)	Spns2 (216892)	Exports S1P to the outside of the cell membrane.	**Inhibitor**: SLF1081851.	Colorectal cancer. Oral squamous cell carcinoma. Lung cancer. Liver fibrosis. Chronic obstructive pulmonary disease.
S1P receptor (S1PR)			G protein-coupled receptor that recognize S1P and SPC. Regulation of biological processes.		
S1PR1	S1PR1 (1901)	S1pr1 (13609)	Binds S1P with high affinity. Gαi/Gαo-, Gα12/Gα13-, and Gαq-coupled. Involved in vascular system development and maintenance. Modulates immune cell distribution and function	**Modulators**: FTY720 (Fingolimod), KRP-203, Syl930, Siponimod, Ponesimod, Ozanimod, AUY954, SEW2871, RP-101075, SAR247799, CYM5442, Amiselimod, Syl930, CS-0777, W061; **Antagonists**: VPC23019, W146, VPC44116, CL2 (chemical lead 2).	**Lymphoma**. **Liver cancer**. **Tumor metastasis**. **Multiple sclerosis**. Glioblastoma. Bladder cancer. Breast Cancer. Gastric cancer. Endometriosis. Lung cancer. Prostate cancer. Gallbladder cancer. Colorectal cancer. Urothelial carcinoma. Esophageal squamous cell carcinoma. Cancer-drug resistance. Graves’ orbitopathy. Autoimmune thyroiditis. Liver fibrosis. Asthma. Atherosclerosis. Sepsis.
S1PR2	S1PR2 (9294)	S1pr2 (14739)	Gs-, Gq/G11-, and G12/G13-coupled. Negative regulation of S1PR1-mediated signaling.	**Agonist**: CYM5478; **Antagonist**: JTE-013, AB1.	**Hearing impairment (Deafness)**. **Lymphoma**. Colorectal cancer. Liver fibrosis. Bladder cancer. Cholangiocarcinoma. Glioblastoma. Oral squamous cell carcinoma. Multiple sclerosis. T2D. Abdominal aortic aneurysms. Endometriosis.
S1PR3	S1PR3 (1903)	S1pr3 (13610)	Gi/Go-, Gq/G11-, G12/G13-coupled	**Modulator**: FTY720; **Antagonists**: VPC23019, KRX-725-II, CAY10444, TY52156, VPC44116.	**Breast Cancer**. Lung cancer. Tumor metastasis. Glioblastoma. Ependymoma. Nasopharyngeal carcinoma. Renal cancer. Bladder cancer. Acute respiratory distress syndrome. Pulmonary edema. Acute lung injury. Sepsis. Multiple sclerosis. Liver fibrosis. Endometriosis.
S1PR4	S1PR4 (8698)	S1pr4 (13611)	Gi/Go-, G12/G13-coupled	**Modulators**: Phyto-S1P, VPC23153, FTY720, KRP-203, W061, Amiselimod; **Antagonists**: JTE-013, CYM50367,	Breast cancer. Obstructive coronary artery disease.
S1PR5	S1PR5 (53637)	S1pr5 (94226)	Gi/Go-, G12/G13-coupled	**Modulators**: FTY720, Siponimod, Ozanimod, W061, Amiselimod.	Large granular lymphocyte leukemia. Glioblastoma. Chronic obstructive pulmonary disease. Multiple sclerosis.
Sphingosine-1-phosphate phosphatase (SPP)			Dephosphorylates S1P to sphingosine		
SPP 1	SGPP1 (81537)	Sgpp1 (20750)	Salvage and recycling pathway for ceramide. Located in the ER and membrane. Modulates apoptotic signaling.	–	Endometriosis. Gastric cancer. Colorectal cancer. Tumor metastasis. Schizophrenia.
SPP 2	SGPP2 (130367)	Sgpp2 (433323)	Located in the ER membrane. Related to the inflammatory response.	–	Psoriasis. Endometriosis.
Sphingosine-1-phosphate lyase (SPL) 1	SGPL1 (8879)	Sgpl1 (20397)	Irreversibly degrades S1P to phosphoethanolamine and hexadecanal. Decreases the level of S1P	**Inhibitors**: Tetrahydroxy-butylimidazole (THI), LX2931, LX3305.	**Adrenal insufficiency**. **Nephrotic syndrome**. Ichthyosis. Alzheimer’s disease. Huntington’s disease. Charcot-Marie-tooth neuropathy. Oral squamous cell carcinoma. Pituitary adenoma. Colorectal cancer. Colon cancer. Prostate cancer. Gastric cancer. Gastroesophageal cancer. Liver cancer. Alveolar rhabdomyosarcoma. Preeclampsia. Endometriosis. Cryptorchidism.
Sphingomyelin deacylase	–	–	Converts sphingomyelin to sphingosylphosphorylcholine (SPC).	–	**Atopic dermatitis**.
Autotaxin (ATX), ENPP2	ENPP2 (5168)	Enpp2 (18606)	Converts lysophosphatidic acid (LPC) and sphingosylphosphorylcholine (SPC) to lysophosphatidic acid (LPA) and S1P, respectively. Increased in an inflammatory response.	**Inhibitors**: PF-8380, BBB-877, ONO-8430506, CRT0273750, HA-130, HA-155, GLPG1690, S-32826.	**Breast cancer**. **Hepatitis C**. **Liver cancer**. **Liver cirrhosis**. **Primary sclerosing cholangitis**. **Insulin resistance**. **Obesity**. **Melanoma**. Preeclampsia. Pregnancy-induced hypertension. Intrahepatic cholestasis of pregnancy. Biliary atresia. Alzheimer’s disease. Systemic sclerosis. Pancreatic cancer. Renal cancer. Thyroid cancer. Tumor metastasis. Type 1 endometrial cancer. Hematological malignancy. High-grade serous carcinoma. Prostate cancer. Esophageal cancer. Colorectal cancer. Glioblastoma. Bladder cancer. Hodgkin lymphoma. Ovarian cancer. Tumor angiogenesis. Cancer-drug resistance. Epstein‒Barr virus infection. Threatened preterm delivery. Asthma. COVID-19. Idiopathic pulmonary fibrosis. Blister. Mild cognitive impairment. Crohn’s disease. Ulcerative colitis. Posner–Schlossman syndrome. Acute myocardial infarction. Nonalcoholic fatty liver disease. Hepatitis B. Cholestasis. Cholestatic pruritus. Primary biliary cholangitis. Type 1 autoimmune pancreatitis.

**Fig. 2:**
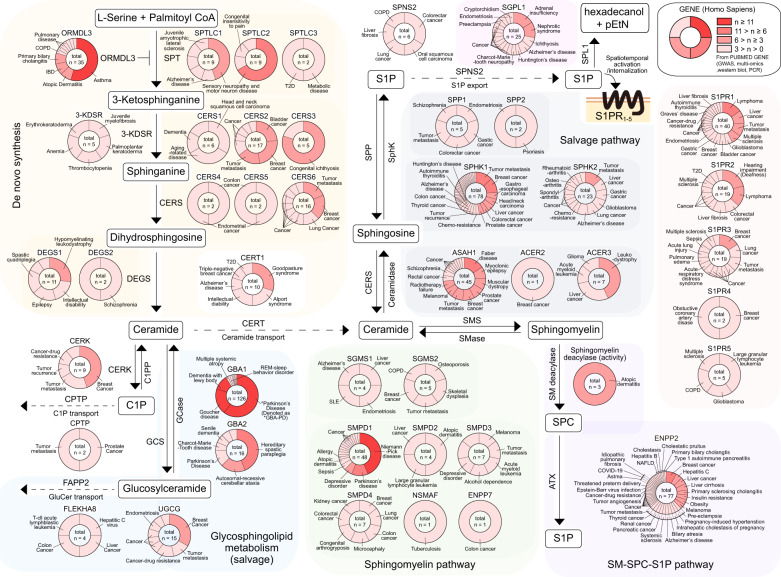
Sphingolipid-related enzymes and reported human disease. Alteration of sphingolipids is closely related to human disease. The numbers of reported human studies that analyzed genome-wide association studies, multiomics-based data, Western blot, and PCR comparison of disease tissue to control tissue were counted as visualized (PubMed Gene). The counted numbers are subdivided by color to facilitate understanding of the roles of sphingolipid-related enzymes in human disease.

Sphingolipid-modulating enzymes and their NCBI gene ID number with their function, modulators, and reported human diseases (Analysis of disease: ~2022. Dec). Related human diseases are referenced from the NCBI GENE database (Bibliography-all citations in PubMed)^21^ with statistically significantly different data from GWAS, multiomics, Western blot, and PCR analysis (bold, reported articles (cases) ≥3). See also Fig. 2.

Reciprocal interference and modulation among sphingolipids are crucial for the immune context because the misguided “appearance” (migration, activation, and clonal expansion) or “exit” (ignorance, senescence, anergy, exhaustion, apoptosis, or transmigration) of immune players in the inflamed tissue stage is closely related to excessive immune responses or immune paralysis^4,16,19,20^. Likewise, numerous research studies that investigated human disease with genome-wide association studies and multiomics analyses of disease tissue compared to healthy control tissue (from the PubMed Gene database^21^) revealed that disrupted sphingolipids and their related enzymes are closely related to hereditary neuronal diseases and life-threatening diseases such as tumors and autoimmune disorders (Table 1 and Fig. 2). These results imply the importance of sphingolipid homeostasis in human health. Investigation of sphingolipids has focused on the fate of cells, namely, survival, proliferation, maintenance, or death, with altered balances among sphingolipids, and these metabolic reactions of sphingolipids are interconvertible and reciprocally communicated^9–15^. Ceramides and S1P are the most studied sphingolipids with respect to the functions of immune cells. Extrinsic signaling cues such as tumor necrosis factor-α (TNF-α) and lipopolysaccharide (LPS) and/or intrinsic modulators such as cytosolic kinases and transcriptional modulators (e.g., Foxp3, a master transcription factor of regulatory T (T_reg_) cells, can transcriptionally inhibit the expression of Sgms1, which converts ceramides to sphingomyelins) can cause ceramide accumulation, and the increased ceramide levels can selectively turn on/off intracellular machinery such as protein phosphatase 2 (PP2A), nuclear factor-κB (NF-κB), and mitogen-activated protein kinases (MAPKs)^4,22–24^. Moreover, enrichment of the intracellular content of ceramides can control the trafficking rate of proteins to the Golgi complex and membrane, which may cause cellular senescence^25,26^. Calcium-dependent activation of CERK can convert ceramide to C1P, and C1P can be secreted into extracellular regions as an auto/paracrine signaling cue or can directly act as an intracellular signaling messenger. While ceramide blocks the PI3K-Akt-mTOR pathway with PP2A activation to modulate cellular functions such as proliferation and survival, C1P enhances PI3K-mediated signaling, cell proliferation, and survival. Moreover, C1P can bind and activate cytosolic phospholipase A_2_, which in turn stimulates the production of arachidonic acid-derived prostaglandins (PGs), and these PGs modulate pro-/anti-inflammatory immune responses in a context-dependent manner^4,25,27^. The opposite roles of ceramide and S1P are also well characterized in cell death/survival and immune responses by modulation of bcl-2 and bcl-2-like protein (Bax) and the subsequent intracellular machinery for programmed cell death^28^, and these sphingolipid-related enzymes are closely related to the pathogenesis and development of tumors (Table 1 and Fig. 2). One significant and interesting factor in the treatment of cancer is that tumor cells can also modulate sphingolipid metabolism with ceramidase, SphK and ATX to favor their survival and maintenance while blocking the patient’s immune system^29,30^. The functional roles of S1P and its receptor (S1PR) in extracellular and intracellular responses have been extensively investigated, and S1PRs are closely related to human diseases such as cancer^4,30^ (Table 1). Understanding the kinetics of sphingolipids and related enzymes may provide insight into the current state of immune cells, and by selectively modulating these molecules (Figs. 1 and 2 and Table 1), we can develop patient-specific medicine suitable for the specific disease status. Fingolimod (FTY720, Gilenya) and its phosphorylated form (phosphorylated by SphK, p-FTY720) are clinically used as treatments to target S1PR_1,3-5_, but not S1PR_2_, for the treatment of autoimmune diseases such as multiple sclerosis (MS) by sequestering lymphocytes in lymph nodes and therefore attenuating the migration of inflammatory lymphocytes into inflamed sites^31^. Likewise, the modulation of sphingolipids and their related enzymes has been studied in the clinic^32,33^, which will be discussed in more detail in the following sections.

## Sphingolipids as prognostic and diagnostic indicators of disease

**Fig. 3:**
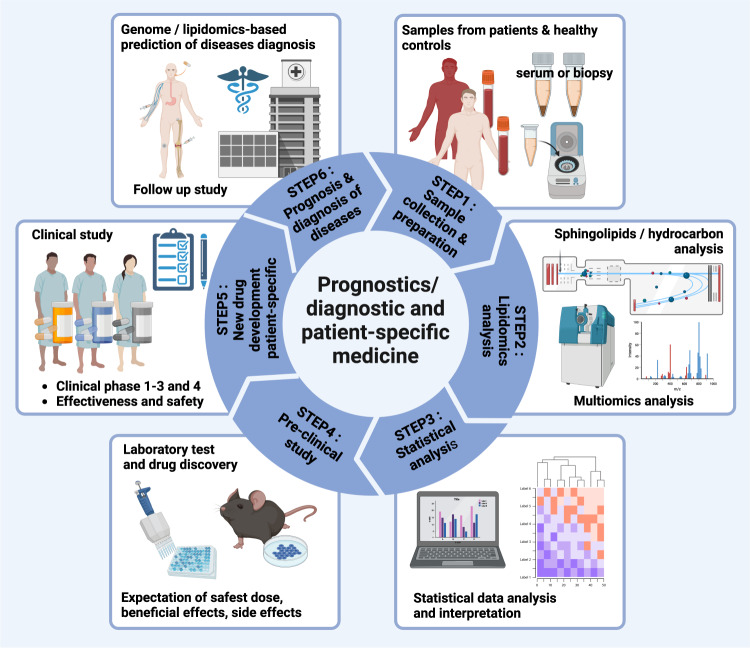
Strategy for diagnosis/prognosis of diseases with sphingolipids. Recent progress in metabolomics and multiomics analysis has enabled the investigation of sphingolipids as a marker of disease. After collection of human blood and disease tissue by biopsy, a series of procedures with mass spectrometry enable the detection of changes in sphingolipids and related molecules compared to levels in healthy volunteers. By analysis of a multiomics database, specific sphingolipids and related enzymes can be detected for human disease. Inhibition of sphingolipid enzymes in a preclinical disease model should enable investigation of the roles of sphingolipids and related enzymes.

Several human cohort studies of diabetes, tumors, autoimmune, neuroinflammatory, and degenerative diseases, among other diseases, have revealed that alterations in sphingolipid metabolites in the blood can be detected several years before/during disease development, and these abnormalities of sphingolipid metabolism and sphingolipid-related enzymes in tissue may be closely related to the progression and pathogenesis of disease^34^ (Table 1 and Fig. 2). Glucosylceramidase beta 1 (GBA1) is a representative example that has been reported to be the etiological factor for Parkinson’s disease (PD), which results from heterozygous mutation of this gene, now categorized as GBA-PD^35^. Enhanced S1P and C1P-producing enzymes (CERK, SphK, and ATX) and activation of S1PRs in tumor tissues are closely related to poor prognostic factors that result in tumor metastasis and recurrence while attenuating chemo- and antitumor therapies, deteriorating the survival of tumor patients^36–39^ (Fig. 2 and Table 1). Analyses of open databases collected from a patient-derived genome atlas can also facilitate the prediction of a relationship between alterations in sphingolipid metabolism and disease progression as a prognostic approach for diseases such as tumorigenesis^25,40^, Alzheimer’s disease^41^, systemic lupus erythematosus (SLE)^42^, and metabolic diseases^43^. Prediction of disease progression in the clinic with a few drops of blood and/or minimalized biopsy followed by pretreatment with prospective patient-specific medicines may be goals for unmet medical needs to improve our medical welfare (Fig. 3). To understand the alterations in sphingolipid metabolism and related enzymes in immune responses and disease, several methodological approaches are now available for the detection/digitization and visualization of sphingolipid contents, as well as during the progression of de novo synthesis/degradation^44–46^. Classically, after extraction of lipids from cells and tissues with high-performance liquid chromatography (or thin-layer chromatography), electron ionization of molecules followed by tandem mass spectrometry-based sphingolipidomic analysis can provide structural and quantitative measurement of sphingolipids regarding molecular weight (mass-to-charge ratio, *m/z*), specific information on the structure (length of N-acyl chains, bases, and composition of head groups), and retention times. Although these techniques cannot distinguish molecules that have geometrical and optical isomers or produce similar ion products and require appropriate stable-isotype controls, the approaches present very sensitive and precise information on the molecules, including quality/purity and quantity. Therefore, researchers can determine the current composition of tissue, which has now been adapted for imaging (mass spectrometry imaging)^46,47^. Analysis of brain tissue from MS patients with phospho-proteomics has revealed distinct phosphorylated protein patterns and has uncovered the uncontrolled S1P-S1PR_1_-mediated T_H_17-triggered immune response that causes enhanced neuroinflammation^48^. Likewise, analysis of mass spectrometry-based techniques has provided links between dysregulated sphingolipid contents and defects in sphingolipid-related enzymes in the pathogeneses of pulmonary disease^49^, inflammatory bowel disease^17^, cancer^30,50^, and other diseases (Table 1), proving the important roles of sphingolipids in human health and disease. Analysis of the in silico retention time by a quantitative structure retention-relationship approach, comparison with a database from a mass spectral library, and/or analytic programs can facilitate the identification of sphingolipids and the interpretation of data from a target sample^51^. Recently, Muralidharan et al. isolated and investigated the composition of 114 different sphingolipids from 21 different murine tissues and plasma using targeted lipidomics^52^. According to the results, only 11 sphingolipid species are common to all tissues, which may contribute to the fundamental and essential functions of tissues, such as cell cycle regulation, while each tissue shows its own distinct distribution of sphingolipids and shows similar patterns among functionally close tissues. Since each tissue has its own specific pattern/composition of hydrocarbons in sphingolipids, analysis of blood can reveal the status of an organ that is expected to be injured or inflamed. Skin, stomach, and intestines have a tendency to have very long-chain fatty acids (C26:0, saturated 26 hydrocarbons), which may relate to barrier function with thicker membranes. Principal component analysis of tissue sphingolipids revealed that immune-related organs such as the spleen, thymus, and lymph nodes are more closely clustered, with a closer cluster between the spleen and thymus, followed by the lymph nodes, compared to the intestine, stomach, lung, brain, and adipose tissue^52^. However, brown adipose tissue is more similar to the lymph node, thymus, and spleen.

Phylogenetically, the majority of bacterial species cannot produce sphingolipids, but *Bacteroides*, one of the most predominant commensal microbiomes in the intestine, can produce and provide a source of ceramides with both odd (C17:0) and even (C18:0) numbers of hydrocarbons, identical to mammalian ceramides, attenuating inflammatory responses and contributing to homeostasis of the intestinal immune system, while host-derived sphingolipids from the intestine aid the maintenance of *Bacteroides* species or function to control their abundance with bactericidal sphingosine^17,18,53^. In contrast, uncontrolled growth of pathogenic bacteria such as *Pseudomonas*, *Staphylococcus*, or *Mycobacterium* can disrupt the balance of the bacterial flora, and these pathogens hijack and impair host sphingolipid metabolism^53^. Interestingly, the spleen contains S1P with an odd number of sphingoid bases C17:1 (17-carbon with 1-unsaturated hydrocarbon chain), which usually cannot originate from mammals but can be produced by the microbiome, along with the plasma, skin, small intestine (but not the large intestine), and lungs of normal C57BL/6 mice, implying that the microbiome can affect and shape the systemic immune response^52,53^. Likewise, uptake of *Bacteroides*-derived sphingolipids (C17:0) can affect the host cell’s de novo synthesis of sphingolipids (C18:0), showing an increased frequency of odd-numbered S1P and ceramide production compared to even-numbered S1P and ceramide^18^. Similarly, in humans, the majority of fatty acid hydrocarbons are even-numbered, and less than 1% of fatty acids are odd-numbered in plasma. It has been reported that increased C15 and C17 are closely related to insulin resistance, and these are now accepted as biomarkers for type 2 diabetes^54^. The results obtained from studies that map the lipid distribution of tissues complement the tissue-specific sphingolipid atlas. Analysis of genomic approaches such as bulk mRNA and single-cell RNA sequencing analyzing lipid-related enzymes and previous reports and databases has suggested another approach for understanding tissue-specific immune (and/or nonimmune) responses in diseases. For example, mass spectrometry followed by network analysis of tumor cells after chemotherapy treatment revealed that chemotherapy of tumor cells or administration of medicine can alter sphingolipid-related enzymes such as SMase, CERK, and SphK and subsequently alter cellular sphingolipids, which may help decipher the malignancy of tumors and drug resistance^38,55–57^. Although these results from inflamed whole tissues may explain the unresolved aspects of the conventional immune response, information on sphingolipids from specific immune cells in normal and disease conditions, classified by the involvement of immune organs such as the spleen, thymus, lymph nodes, and bone marrow, should be investigated and discussed in more detail.

The development of traceable fluorescent molecules enables us to chase and investigate the de novo synthesis, hydrolysis, and transfer of sphingolipids with equipment for fluorescence microscopy, flow cytometry, and mass spectrometry. Incorporation of azide-functionalized, or other photoswitchable, sphingolipids followed by application of biorthogonal click chemistry, which is selective for the azide-/alkyne-modified region, with BODIPY usually conjugated as fluorescence, can reveal the alterations in the sphingolipid content and visualize the location of tagged sphingolipids^58–60^. Super-resolution microscopes such as expansion microscopes or atomic force microscopes can provide an alternative approach for sphingolipid imaging^60,61^. Quantification of sphingolipid-related mRNA and protein expression under certain conditions and subsequent gain-/loss-of-function study of a specific gene after biopsy can further strengthen the understanding of the roles of sphingolipids in normal/disease states. Techniques developed for analyzing sphingolipid metabolism, antibodies that detect sphingolipid-related enzymes and receptors, and sphingolipid-related transgenic or knockout (KO) mice in preclinical studies can be utilized as diagnostics for disease and may lead to the development of new drugs for clinical studies. Collectively, investigation of sphingolipid metabolism may supplement our understanding of the immune system in normal homeostatic and disease states, which previously could not be fully explained by nucleotides and proteins alone.

## Sphingolipids as warning molecules and modulators of the innate immune response

**Fig. 4:**
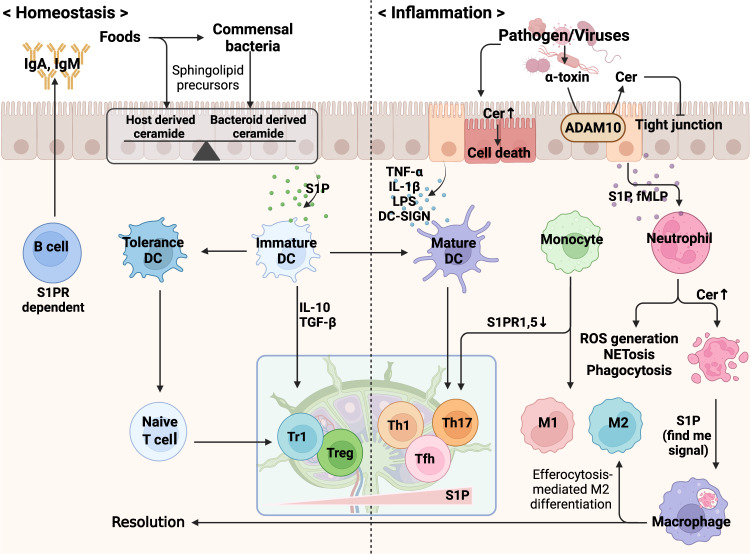
Homeostasis and immunological functions of sphingolipids. Sphingolipids can be derived from the host and food metabolites and can be modulated by interactions with commensal bacteria, maintaining immune tolerance. Infection with pathogenic bacteria can block and hijack host sphingolipid metabolism, activating immune cascades. Pathogens can stimulate epithelial cell death and enhance the contents of ceramides that facilitate the breakdown of the barrier. Innate immune cells such as neutrophils and macrophages detect inflammatory cues and migrate into inflamed sites. Increased intracellular and extracellular sphingolipids guide the direction of the immune response, and pathogens can attenuate the host immune response, while antigen-presenting cells activate the adaptive immune system. S1P, as a ‘find me’ signal from apoptotic cells, induces resolution with an efferocytic process.

Deviation from mutual competition or alliance of the microbiome with the host defense system leads to the direction/flow of ceramide metabolism, and the leading entity that possesses control of the host machinery determines the context of the immune response^17,53^. The phylum *Bacteroides*, containing members such as *Bacteroides*, *Parabacteroides*, *Prevotella*, and *Porphyromonas*, which compose the majority of the human gut microbiome, can process food-derived metabolites and supply a source of sphingolipids to epithelial cells, which can eventually be absorbed by the hepatic portal vein and the circulatory system^18,62^. With these absorbed sphingolipids, commensal bacteria can affect and govern the host metabolic system and immune response. The most differentially abundant metabolites in the stool of patients who suffer from inflammatory bowel disease, such as ulcerative colitis and Crohn’s disease, are host-produced ceramides, sphingomyelins, and sphingosines (C18:1), while sphingolipid-producing bacteria and *Bacteroides*-derived sphingolipids are significantly decreased. The study of SPT-sufficient or SPT-deficient *Bacteroides thetaiotaomicron*-adopted mice revealed the crucial role of commensal-derived sphingolipids in intestinal immune homeostasis^17^. Although commensal bacteria provide a source of ceramides and attenuate excessive intestinal inflammation, by crossing the barrier and subsequently activating the innate/adaptive immune system, the excessive “Renaissance” of commensal bacteria can also trigger activation of the host immune system, especially humoral immunity, with the production of antibodies and sphingosines that counteract potential sources of danger, ultimately preventing the disruption of the microflora and preparing a healthy host defense system^17,63,64^. In contrast, bacterial pathogens try to neutralize the host defense system and then bind to host epithelial cells and/or secrete bacterial molecules (e.g., toxins) that stimulate the accumulation of ceramides and block the production of sphingosines, facilitating and promoting bacterial colonization^53,64,65^. Although epithelial cells of the mucosal barrier constitute the front line of physical defense and are coated with mucins, defensins, IFN-λ, and antibodies IgA and IgM^66–68^, when the defense wall is damaged, epithelial cells request the reinforcement from innate/adaptive immune cells with chemokines and sphingolipid S1P^69^. The functional roles of S1PRs have been investigated by analyzing the spatiotemporal distribution and internalization of receptors in response to sphingolipids^69,70^. Here, we briefly review the recent understanding of immune cells from the aspect of sphingolipid metabolism (Fig. 4).

Neutrophils, the most abundant and crucial defenders of the innate immune system, patrol the bloodstream, and when they detect danger signals with pattern recognition receptors, they migrate into inflamed sites and do their jobs in a context-dependent manner^19^. The decision for eternal sleep (apoptosis) or not (NETosis, necrosis, and ferroptosis) after their role is complete is crucial because a neutrophil corpse that is left behind can act as a “dying message” to other immune cells that can follow the intention of the deceased cell. Not surprisingly, from the cradle (granulopoiesis) to the grave, sphingolipids can tremendously affect a neutrophil’s short life^19^. When human CD34^+^ hematopoietic stem/progenitor cells are activated in vitro, the TNF-α-triggered neutral SMase (nSMase)-ceramide axis can shape the hematopoietic system to favor myelopoiesis but not erythropoiesis, while S1P can restore erythroid differentiation, switching the direction of GATA, PU.1, and the autophagy-related machinery^71^. S1P and fMLP secreted from inflamed epithelial cells and tissues can attract the migration of neutrophils, and the intracellular ceramide content of neutrophils can be increased when TNF-α and fMLP are encountered^72,73^. Increased ceramide can modulate the function of neutrophils in terms of cell migration, the generation of reactive oxygen species, neutrophil extracellular trap formation, and bactericidal activities^74–77^, and increased sphingomyelin in neutrophils may be related to neutrophil infiltration and phagocytic activity^78^. Likewise, CerS2,6 modulates the migration of neutrophils, and CerS6 KO exacerbates the development of experimental autoimmune encephalomyelitis (EAE) with enhanced infiltration of neutrophils, while CERS2-deficient mice showed delayed development of EAE with decreased chemokine receptors (CCR1, CXCR1, and CXCR2)^79,80^, implying that the length of the carbon chain attached to the sphingoid base may differentially affect neutrophil function. One of the most interesting aspects of neutrophil-mediated phagocytosis is that this engulfment can increase the expression of SMase and subsequent ceramide production, and administration of exogenous ceramide can inhibit neutrophil functions such as phagocytosis and degranulation, while C1P can enhance phagocytosis^77,81,82^. Upon exposure to immune contexts such as fMLP, SphK activity can be increased in neutrophils, and the S1P that is subsequently produced can enhance/sustain neutrophil function in an autocrine or paracrine manner. Likewise, SphK1 deficiency in neutrophils results in diminished pathogen-killing activity, and administration of exogenous S1P rescues the function of SphK KO neutrophils. The accumulation of ceramide (de novo, C16- and C24-ceramide) can turn on programmed cell death in neutrophils^83^, and the apoptotic body can initiate resolution with the efferocytosis process, educating immune-suppressive innate/adaptive cells^19^. However, exposure to S1P, as well as IL-8 and HMGB1, can induce the expression of anti-apoptotic Bcl-x in neutrophils, and prolonged exposure to these inflammatory cues can induce other forms of neutrophil death, such as necrosis or ferroptosis, followed by “frustrated” efferocytosis, which are closely related to chronic inflammatory diseases^19^.

During/after immune responses, neutrophils can request reinforcement from other immune cells, such as monocytes, macrophages, and dendritic cells (DCs), to inflamed tissues^19^. The spatiotemporal action/activation of ceramide, C1P, S1P-S1PR, and related surface receptors such as CD69 can guide the distribution of these innate immune cells and orchestrate the overall immune response, while these cells can be a source of sphingolipids such as S1P^4,16,73,84^. During immune responses, exposure to TNF-α activates CERK/SphK and stimulates the phosphorylation of ceramide/sphingosine, resulting in the production of the proinflammatory cytokines IL-1β and CCL2^85,86^. During the progression of murine MS, activated inflammatory monocytes (CD11b^+^CCR2^+^Ly6C^high^Ly6G^low^) can migrate to and temporally reside in lymph nodes with expression of CD69, which retains inflammatory monocytes in the lymph node by internalization of S1PR_1,5_, and these monocytes prime and educate lymphocytes by supplying S1P and inflammatory cytokines for the development of T_H_17 and follicular helper T cells^84^. The function and phenotype of monocytes are heterogeneous and plastic in immune responses, and monocytes can differentiate into proinflammatory (M1) or immune-regulatory (M2) phenotype macrophages or DCs, which have distinct roles^87^. S1P-S1PR_1_ mediates the trafficking and migration of monocytes and macrophages^88^, and S1P can block the apoptosis of macrophages via PI3K-Akt signaling while skewing phenotype switching toward M2^89^. S1PR_2_ can oppose S1PR_1_-mediated migration and differentiation of macrophages, with S1P-S1PR_2_ altering macrophage phenotypes to M1 and suppressing phagocytosis but not bactericidal capacity^89–92^. TNF-α activates CERK and stimulates C1P production, which can modulate the induction of a proinflammatory phenotype in macrophages, and secreted C1P directly inhibits TNF-α converting enzyme (TACE/ADAM17, the active form of which resides within the cholesterol-rich membrane region) and therefore calms down the LPS-induced production of mature TNF-α, cutting off the excessive cycle of inflammation^85,93^. One of the main immunological roles of monocytes and macrophages is “to eat”, i.e., phagocytosis, which is the clearance of pathogens, foreign molecules, cellular debris, and apoptotic bodies^94^. The synthesis of sphingolipids, but not glycosphingolipids, is required for the phagocytosis of specific pathogens such as *Mycobacterium tuberculosis*, thereby modulating actin dynamics and the formation of the phagocytic cup during engulfment and internalization^95^. Analysis of phagocytosis with quantitative lipidomics during the process of phagosomal maturation revealed that early phagosomes have enhanced activity of neutral ceramidase compared to CerS under neutral (pH 7.0) conditions, resulting in decreased ceramide levels. However, ceramides and glucosylceramides with long hydrocarbon chains (16:0, 18:0, 20:0, 22:0, and 24:0 but not 12:0 and 14:0) are gradually enriched on mature phagosomes of Raw264.7 cells, a murine macrophage cell line, with enhanced glucosylceramide synthase (GCS) activity, while sphingosines and the expression/activity of CerS2 are decreased^96^. During phagosomal maturation, the levels of sphingomyelin and C1P are comparable in early and late phagosomes, but activation of CERK and C1P can facilitate Fc receptor-mediated phagocytosis, collectively implying the delicate roles of sphingolipids in macrophage function^82,96^. Although some bacteria and hosts have evolved to live together by symbiosis, other bacteria called pathogens have evolved to exploit and hijack host metabolites for their own purposes^53,62^. The sphingolipid metabolism pathway is not exempt from being hijacked, and some pathogens cause the disruption of host sphingolipid metabolism to neutralize and counteract the host defense system^53,62^. Different pathogens utilize different strategies; fungi synthesize their own sphingolipids and specific enzymes for synthesis and can use their own and host sphingolipids. Most pathogenic bacteria and viruses act as “parasites” of host sphingolipid metabolism, thereby disturbing the host defense system^16,53,65^. *Neisseria gonorrhoeae*, the major cause of meningitis and septicemia, *Pseudomonas aeruginosa*, which is an opportunistic pathogen and the cause of cystic fibrosis and sepsis, and *Staphylococcus aureus* can stimulate the acid SMase (aSMase) of epithelial cells and fibroblasts, facilitating invasion through a sphingomyelin-decreased and ceramide-enriched platform^97–99^ and evading the innate immune defense system^100–102^. The enrichment of ceramides in host cells can induce cell death and thereby destroy the barriers formed by cells. Even worse, several kinds of toxins produced by pathogens (e.g., the α-toxin produced by *S. aureus*) can bind to surface metalloproteinases (e.g., ADAM10) of the host and induce the release of acid ceramidase-mediated ceramide outside of the inflamed walls, which eventually leads to degradation of tight junctions^99^. Moreover, the produced chemokines and S1P recruit innate/adaptive immune cells, and recruited macrophages can wisely utilize the SMase/ceramidase strategy of the pathogen to rearrange its cytoskeleton, facilitating the process of phagocytosis^96,103^.

A series of fierce battles end with dead immune and tissue cells, and upon completing their duties, some immune cells, such as neutrophils, accept their programmed death, apoptosis, in the immune context to herald the end of the war and a new era of regeneration^19,104^. Phagocytes can trace CX3CL1, ATP, LPC, and S1P as “find me” signals from apoptotic bodies, and apoptotic body-derived S1P can turn on the anti-apoptotic system of macrophages and stimulate the production of anti-inflammatory IL-10 to decrease inflammatory responses^104^. Macrophages check the state of the target to determine whether it has exposed phosphatidylserine on the cell surface to distinguish dead targets from live targets, which express CD47, CD31, and CD24 as “don’t eat-me” signals on the outer membrane. During the process of efferocytosis, phagocytes recognize phosphatidylserine with surface receptors such as TIM1, 4 and LDL-receptor-related protein 1, and other receptors such as MERTK and scavenger receptor (CD36) can facilitate the uptake of dead cells and debris^104^. CD36 is a major scavenger receptor for oxidized LDL (oxLDL), and in turn, oxLDL enhances CD36 expression by lipid peroxidation with peroxisome proliferator-activated receptor (PPAR)-γ, and oxLDL can also maintain macrophage survival and enhance proliferation^105,106^. The PPARγ agonists thiazolidinediones can upregulate S1PR_1_^107^, and silencing of aSMase can enhance the transcription of PPARγ-coactivator-1 α (PGC1-α)^108^, which can repress foam cell formation and atherosclerosis^109^. On the other hand, soluble SMase secreted by macrophages and endothelial cells and the subsequently increased ceramide levels are closely related to aggregated atherosclerotic lesions, and ceramides can reduce CD36 expression and thereby decrease oxLDL uptake by monocytes and macrophages^110,111^. The administration of bacterial SMase to macrophages can cause intracellular trapping of CD36, resulting in a significant reduction in CD36 on monocytes and macrophages^110^. Increased ceramide levels via de novo ceramide synthesis can inhibit the efferocytic function of resident alveolar macrophages^112^, while de novo synthesis of ceramides for inflammasome-mediated inflammation is dispensable^113^.

During phagocytosis, macrophages can load some lysosomal particles to the major histocompatibility complex (MHC) and present it as a molecular pattern to lymphocytes, which recognize and initiate the adaptive immune response. Furthermore, macrophages do not present efferocytic particles, if not infected, on the MHC and thereby context-dependently turn on/off the link to the adaptive immune response^19^. DC is another type of potent and professional antigen-presenting cell that directs/maintains the immune context-dependent activation and differentiation of lymphocytes^114^. Before exposure to pathogens, DCs reside in peripheral tissue in the immature state, waiting for their designation. S1P can promote the migration of human immature DCs but not LPS-stimulated mature DCs^115^, and selective recruitment of immature DCs to inflamed peripheral tissues by S1P implies that S1P favors the role of immature DCs in peripheral tissues in certain immune contexts^114^. Immature DCs can migrate into inflamed sites and then capture and process antigens, thus proceeding to their irreversible maturation process^26,114^. Inflammatory cues such as TNF-α, IL-1β, and LPS and ligation of DC-SIGN and CD40 can stimulate SMase, subsequently leading to the accumulation of intracellular ceramides during DC differentiation, and ceramides are required for pathogen uptake and phagosome formation^26,116^. Moreover, increased intracellular ceramide interferes with vesicle trafficking of newly synthesized or phagocytosed MHC epitopes to be loaded on the MHC, therefore limiting the variation and loading of presented epitopes^26,117^. During maturation, DCs reduce their capacity for antigen uptake and processing while fully optimizing their antigen presentation ability with enhanced expression of MHC and costimulatory surface molecules such as CD80/86, CD83, CD40, and chemokine receptor CCR7 (which leads cells to T-cell zones of secondary lymphoid organs according to gradients of CCL19/21 through lymphatic vessels), and once migrated to the T-cell zones of secondary lymphoid organs, mature DCs efficiently transmit the processed molecules on MHC for epitope-specific lymphocytes to prime/initiate the adaptive immune response^114,118^. Programmed cell death, or apoptosis, of DCs is a physiologically required phenomenon for the elimination of end-stage matured cells. Endogenous ceramides can suppress Akt- and NF-κB-mediated signaling as well as Bcl-x when limited by extracellular cues such as an absence of serum and endogenous and exogenous S1P^119–121^. Inhibition or deficiency of ceramidase or SphK can sensitize DCs to ceramide-induced cell death, and tumor cells such as B16 melanoma release C16/24 ceramides and stimulate ceramide-induced apoptosis of DCs, attenuating DC-mediated lymphocyte activation^120–122^. Collectively, the results imply that the extracellular and/or intracellular contents of sphingolipids and their plasticity in innate immune cells are closely related to the direction of current and subsequent immune responses. Recently, analysis of plasma metabolomics has shown that after anti-tuberculosis BCG vaccination, the levels of sphingolipids such as S1P, sphingomyelin, N-acylsphingosine, and glucosylceramide are significantly changed, and the trained immunity response may be closely associated with these altered sphingolipid metabolites^123^, implicating the involvement of sphingolipids in trained immunity.

## Functional roles of sphingolipids in lymphocytes

The functional roles of sphingolipids, especially ceramides and S1P, have been well investigated in terms of T-lymphocyte distribution and activation^69,118^. The differentiation of each lymphocyte in inflammatory circumstances and in homeostasis are also well characterized with regard to the understanding of immune disease pathogenesis^7,118^. The functional roles of S1P have been characterized with its receptors S1PR_1-5_, and S1P-S1PR regulate many aspects of immune cell function, such as the migration and maintenance of lymphocytes. S1PRs are differentially expressed in lymphocytes; S1PR_1_ and S1PR_4_ are mainly expressed by T cells, while B cells express S1PR_1-4_, but S1PR_3_ is not expressed in human B cells. S1PRs are not necessarily expressed simultaneously and are differentially expressed during diverse stages of cell activation and maturation^7,69,118^. The best studied S1PR is S1PR_1_, with a special focus on lymphocyte migration out of the thymus and secondary lymphoid organs into the blood and inflamed sites, with counteraction of the chemokine receptor CCR7. Spontaneous activation of S1PR_1_ in the lymph node and spleen is required to sustain and maintain naive lymphocytes in normal homeostasis, supplying necessary substances for lymphocytes such as IL-2^124^. S1PR_1_ is the representative receptor that modulates immune cell trafficking and development, stimulating the PI3K-Akt-mTOR and Stat3 pathways, while S1PR_2_ can counteract S1PR_1_-mediated activities^118,125^. Sphingolipid-mediated responses are not stereotypically fixed in one direction of cellular function. For example, by signaling through the differentially expressed S1PRs that can be internalized and/or altered by extracellular and intracellular signaling, on immune cells such as immune-activator conventional T (T_conv_) and T_reg_ cells, S1P can selectively and properly modulate the migration, proliferation, differentiation, and pro-/anti-inflammatory function of immune cells via different S1PRs according to environmental cues. Notably, excessive S1PR_1_ blocks T_reg_ development and function via the Akt/mTOR-mediated pathway and modulates T_reg_ distribution in the body by selective activation with CCR7, directing time-dependent modulation of early-time immune activation of T_conv_ cells followed by the immune-regulatory response of T_regs_ and affecting development of resident memory T cells that migrate to the lymph nodes^126,127^. Likewise, excessive S1PR_1_ activation, which activates the PI3K-Akt- and p-Stat3-mediated T_H_1 and T_H_17 responses, and enhanced expression of S1PR_1_ have been reported in autoimmune patients with MS^48,128^. S1P can enhance TNF-induced expression of the receptor activator of nuclear factor kB ligand and in turn aggravate the pathogenesis of inflammatory bone disease^129,130^. Due to its similar structure to other sphingolipids, especially S1P, the signaling pathways of SPC have been investigated with regard to S1PRs, and the study of the roles of SPC has focused on vascular/cardiovascular disease and tumors^131^. Studies of fingolimod, a novel functional antagonist for S1PR_1,3-5_, have revealed and supported important roles of S1P-mediated signaling in the modulation of immune disease. In an unphosphorylated or phosphorylated form mediated by SphK, fingolimod can impair the ability of lymphocytes and induce the internalization and terminal degradation of S1PR_1_, a crucial receptor for lymphocyte maintenance, proliferation, and function as well as distribution in the host body^132^. Ozanimod is a newly FDA-approved medicine for MS that selectively antagonizes S1PR_1,5_, and several S1PR modulators have been developed for the selective inhibition of S1PR^133^. Likewise, modulation of sphingolipids and their related enzymes has been studied in the clinic. Inhibition of tumor sphingolipid metabolism with fingolimod or fenretinide facilitates the apoptosis of tumors while blocking the cell cycle and survival of cancer cells^134^. Although SPC and S1P can share the same S1PRs, SPC and S1P oppositely regulate the activity and expression of ATX (Enpp2), which is positioned between SPC and S1P in the SPC-ATX-S1P axis, and as such, the choice of SPC and S1P in S1PR signaling may be another modulator of cellular responses similar to LPC and LPA^14,15,131^. In a normal immune system response, S1PRs are differentially internalized or enhanced with context-dependent activation of lymphocytes, according to environmental cues. Ceramide is another important player in lymphocyte activation. Of note, T-cell receptor (TCR)-induced ceramides can serve as a negative regulator of TCR signaling and can modulate the activation, survival, and proliferation of lymphocytes with IL-2 secretion and the distribution and signaling of TCR as nanoclusters^135–137^. Likewise, coreceptor CD28 engagement can enhance SMase-mediated ceramide generation with PI3K-Akt-mTOR signaling that promotes lymphocyte activation and proliferation^138^ and modulates the transport of IL-2^139^, collectively implying the crucial roles of sphingolipids in mediating TCR signaling. Likewise, functional roles of SMase in T_H_1, T_H_17, and T_reg_ cells were reported^140^. With an increase in SMase activity and increased ceramide production, ceramides enhance T_H_1/T_H_17 differentiation via phosphorylation of JNK and PI3K-Akt-mTOR^140,141^, while CD28-mediated activation of SMase acts as a negative regulator of T_reg_ function and differentiation, dampening the induction and stability of the T_reg_-specific transcription factor Foxp3^142^. However, intermediate TCR stimulation triggered by an adequate amount of antigen can induce FOXP3 expression, and T_regs_ contain high levels of ceramides for PP2A activity that modulate IL-2Rβ signaling pathways and sustain T_reg_ stability by trapping its modulatory protein SET^22,143^, implying that the proper level of ceramides and the modulation of related intracellular enzymes and molecules are important for lymphocyte fate. Differentially expressed PP2A in T_reg_ and T_conv_ cells (with high expression of SET in the resting state) results in the high sensitivity to IL-2 of T_regs_, which cannot express IL-2, and therefore facilitates prior activation of T_regs_ compared to NKT, CD8, and CD4 T_conv_^144^.

Sphingolipids in cytotoxic lymphocytes are now being looked at with interest to understand the modulation of immune responses against viruses or tumors^145,146^. Encountering a virus can trigger activation of SphK1/2, which modulates clonal activation of CD8 T cells to control viral clearance and persistence. Although SphK1 and SphK2 share the same enzymatic function of S1P generation, SphK1 mainly localizes to the cytosol and plasma membrane, while SphK2 resides in the nucleus and cytoplasm. In an interesting viral response, viruses hijack host SphK1/2 to replicate themselves while suppressing host defense systems^145^. Inhibition of SphK restores and enhances the function of CD8 and CD4 T cells, but SphK deficiency results in excessive lymphocyte numbers and ultimately host death^145^. Likewise, tumor cells activate SphK and ATX to modulate sphingolipid metabolism, favoring an immunosuppressive response in the tumor microenvironment^29,146,147^. Cancer cells utilize the sphingolipid contents to relieve hypoxia- and energy-induced stresses and enhance their survival, promoting autophagy, proliferation, and migration and triggering angiogenesis. Likewise, the functions of ceramide nanoliposomes as antitumor agents are being investigated for the treatment of cancer^32,148^. Collectively, these results imply that sphingolipids can act as immune context molecules presented by viruses and tumors to deceive cytotoxic lymphocytes, and altered sphingolipid metabolism is required for evasion of the host immune system, resulting in exhaustion and anergy of CD8 T cells.

S1PR_1_ and S1PR_2_ regulate B cells in the germinal center reaction and development of memory B cells and plasma cells^149,150^. Aged *S1pr2-*deficient mice develop diffuse large B-cell lymphoma with increased germinal center B cells and spontaneous germinal center formation. Under homeostatic conditions, S1PR_2_ antagonizes the activation of Akt and prosurvival signals, which can be activated by other S1PRs, while S1PR_1_ directs the exit of follicular B cells from the marginal zone to the blood^151^. Collectively, competition to control sphingolipid metabolism among the host, commensal bacteria, and pathogens can decide the direction of the immune response, and therefore, understanding the alterations in sphingolipid signaling in the host immune system and subsequent effective treatment of medicine can facilitate the prevention and treatment of human disease.

## Disrupted homeostasis of sphingolipids in disease

As discussed above, abnormalities in sphingolipid metabolism and sphingolipid-related enzymes are closely related to disease progression (Fig. 2), and these alterations in sphingolipid-mediated signaling in immune players can contribute to the pathogenesis of disease. Considering the crucial roles of sphingolipids in the fate of immune and nonimmune cells as well as inflammagen-like pathogens and tumors, understanding sphingolipid dynamics in the disease-specific immune response may be helpful for controlling human disease. Here, we briefly discuss some studies of how altered sphingolipid metabolism worsens health problems.

### Cancer

Sphingolipids and sphingolipid-related enzymes are strongly related to the development and malignancy of human cancer, and ceramide and S1P have been implicated in tumor immunology^30,134^. With enhanced SphK and ATX and downregulated S1P lyase in tumors, S1P can promote Stat3- and Akt-mediated tumor cell growth with upregulation of Bcl-2/Bcl-xL while resisting p53-mediated apoptosis and stimulating a vicious cycle of tumorigenesis^152–156^. Likewise, downregulation of CerS promotes the development of tumors with prolonged inflammation and dysregulated ER stress^157,158^, and the acidic tumor microenvironment can activate aSMase and induce metalloproteinase-9, which promotes metastasis and immune evasion of tumors^159,160^. Modulation of nSMase2 can enhance the antitumor response to anti-PD-1 therapy^161^, but tumor-infiltrating T_regs_ can reduce the expression of endothelial nSMase2, preventing lymphocyte migration;^162^ moreover, enriched S1P and LPA in the tumor microenvironment can reprogram the antitumor activity of infiltrated lymphocytes^29,163^. The results suggest that targeted sphingolipid modulation in tumor patients can provide a strategy for cancer therapy and can promote the survival of tumor patients^151^.

### Inflammatory disease

Alterations in ceramide and C1P levels can tune the direction of the inflammatory cascade by modulating the TNF-α-mediated response^164^. Deficiency of CerS2 enhances the activity of TACE, thereby worsening LPS-mediated septic shock^165^, while CERK modulates the production of TNF-α by inhibiting TACE^93,166^. Likewise, Cerk-deficient mice showed an impaired immune response to *Streptococcus pneumoniae* with neutropenia, and acid ceramidase loss in myeloid cells attenuated the intestinal recruitment of neutrophils^167,168^. Increased activity of SMase is related to host cell death and organ failure in severe septic patients^169^, and aSMase can paralyze the immune response against *Pseudomonas aeruginosa*, inducing macrophage apoptosis with redox signaling^100^. TNF-α can induce aSMase and subsequent Bid-mediated caspase-3/-9 activation, which can induce cellular apoptosis;^170^ however, aSMase is required for early host defense against *Listeria monocytogenes*^171^ and modulates LPS-palmitic acid-amplified inflammatory signaling in macrophages^172^. Inhibition of SphK1 attenuates the symptoms of polymicrobial sepsis^173^, and deficiency of SGPL1 enhances the proinflammatory response while impairing neutrophil trafficking^174^, implying the differential roles of sphingolipids in the host defense system. Modulation of sphingolipid-related enzymes has also been implicated in intestinal immune homeostasis. Deficiency of CerS6 or CERK aggravates the pathogenesis of experimental colitis^175,176^, and deficiency of alkaline SMase also enhances dextran sulfate sodium-induced colitis in mice with ATX upregulation, the last of which can enhance the T_H_17 and B-cell response in the colon^177–179^.

### Autoimmune disease

FTY720, an FDA-approved S1PR modulator for treating MS, provides proof that the selective modulation of sphingolipid metabolism and its signaling pathway can cure patients with autoimmune disease^31^. Although the differences in the carbon chains in sphingolipids such as CerS2 and CerS6 may affect the progression of autoimmune disease through neutrophil trafficking^79,80^, targeting sphingolipid-related enzymes and sphingolipid receptors is a good strategy for the treatment of autoimmune disease^4^. Knockdown of Cerk ameliorates MS-like behavior and cuprizone-induced demyelination^180^, and genetic deletion of ATX in CD11b^+^ myeloid cells and deficiency of aSMase attenuated the severity of EAE^181,182^. In the progression of SLE, SMPD1 can enhance BCR signaling in B lymphocytes^183^, and the dysfunction of SMPD3 can enhance the inflammatory response of macrophages and B lymphocytes^184^. Pharmacological inhibition of aSMase reduces joint swelling and the production of inflammatory cytokines in antigen-induced arthritis^185^, and increased ATX expression in synovial fibroblasts mediates the pathogenesis of autoimmune arthritis^186^. Likewise, CerS and SMase modulate the lymphocyte allogenic response and impact graft-versus-host disease^187,188^, collectively implying that the inhibition of CERK, SMase, or ATX can be good targets against autoimmune disease.

### Neuronal disease

The most extensively investigated sphingolipid in neuronal disease is Parkinson’s disease- and Goucher disease-associated GBA1^35^. GBA1 modulates the ratio of α-synuclein tetramer-monomer, preventing the accumulation of lipid-rich aggregates and preserving subsequent motor and cognitive function^189^. Haplodeficiency or homozygous deficiency of GBA1 can impair cellular bioenergetics associated with mitochondrial dysfunction and defects in mitophagy^190^. T_H_1- and T_H_17-mediated immune responses are increased, and complement-derived glucosylceramide accumulates in tissue^191,192^. It has been reported that aSMase-deficient mice show a broad range of abnormalities in the central nervous system^193^, and aSMase can modulate autophagic processes in Alzheimer’s disease by modulating lysosomal biogenesis^194^. Neuronal SphK1 can acetylate COX2^195^, which can metabolically produce PGs and mediate the reciprocal activation of amyloid-β and IL-1β in Alzheimer’s inflamed sites^196^, and neuronal-specific deficiency of S1P lyase can aggravate the calcium-dependent hyperphosphorylation of Tau protein and elevate abnormal histone3/4 acetylation pathogenesis and abnormal histone acetylation^197^. The S1P transporter Spns2 is also closely related to LPS- and amyloid-β-induced neuronal inflammation^198^, and the inhibition of SMS1 can be a good approach to controlling Alzheimer’s disease by promoting lysosomal degradation of BACE1^199^. From the early stage of Huntington’s disease, biopsies of patients show increased SGPL1 and decreased SphK1 in postmortem brain tissue^200^, and SphK1 stimulation can exert neuroprotective effects in a mouse model of Huntington’s disease^201^.

### Respiratory disease

Impaired sphingolipid synthesis and/or increased ceramide can induce airway hyperreactivity and allergic response^202,203^. ORMDL3, as a negative regulator of de novo sphingolipid biosynthesis, attenuates antigen- and FcεRI-stimulated mast cell activation by modulating autophagy activation^204,205^, and CD4-specific CerS2 null mice are protected from ovalbumin-induced asthma^206^. Likewise, an accumulation of ceramide can cause pulmonary inflammation and mediate lung fibrosis^207^, and deficiency of aSMase and sufficiency of acid ceramidase can attenuate lung inflammation and fibrosis^208,209^, suggesting that this may be a therapeutic target against respiratory diseases such as chronic obstructive pulmonary disease, asthma, and idiopathic pulmonary fibrosis.

### Metabolic disorder

Ceramide and ceramide derivatives are emerging modulators of metabolic fitness. Hypothalamic neurons of the central nervous system can regulate body weight and energy homeostasis by interacting with the leptin receptor by modulating the expression of GCS^210^. Ceramide is reported to induce vascular dysfunction in diet-induced obesity by dephosphorylation of the eNOS/Akt/Hsp90 signaling complex with PP2A, and inhibition of ceramide synthesis can enhance the insulin response^211,212^. Likewise, inhibition of ORMDL3 impairs adipocyte thermogenesis and induces insulin resistance^213^, and CERT is involved in muscle insulin resistance^214^, while knockdown of Cerk can improve glucose intolerance by attenuating MCP-1/CCR2-mediated inflammation^215^. Alterations in sphingolipid metabolism also modulate the fate of adipocytes and hepatocytes. Defective expression of DEGS1 impairs adipocyte differentiation, and ATX can suppress brown adipose differentiation while enhancing the expression of adipose tissue and impairing the insulin response in diet-induced obesity^216–218^.

Abnormal accumulation of unfolded protein, which induces ER stress, can upregulate de novo sphingolipid synthesis, and ceramide can induce hepatic insulin resistance by suppressing PPARγ2^219,220^. Hepatocyte-specific CerS2 deficiency enhances insulin sensitivity and attenuates diet-induced hepatic steatosis^221^, and overexpression of aSMase in the liver can improve hepatic glucose and lipid metabolism through activation of Akt, GSK3, and AMPK^222^, suggesting the crucial roles of sphingolipids in the metabolic response. The functional role of sphingolipids is also implicated in metabolic liver disease. The expression and activity of SphK1 are significantly increased in fibrotic livers compared to normal livers, and SphK1 can promote liver fibrosis by modulating collagen deposition and α-SMA^223^. Notably, hepatocyte-secreted ATX can aggravate nonalcoholic fatty liver disease by autocrine inhibition of the PPARα/FGF21 axis^224^. On the other hand, inhibition of aSMase can prevent the progression of the early stage of nonalcoholic steatohepatitis^225^, collectively suggesting that sphingolipid-related enzymes can be therapeutic targets against metabolic disease.

## Concluding remarks

Studies on the functional roles of sphingolipids and alterations in sphingolipid contents have been limited due to the limited techniques for the detection of sphingolipids and methods for extraction. Recent progress in lipidomics of the plasma and organ tissue has revealed that changes in sphingolipid metabolites and related small molecules are closely related to human diseases (Table 1 and Fig. 2). This finding implies that analysis of sphingolipid patterns with a few drops of blood and tissue biopsy can be used as diagnostic and prognostic tools (Fig. 3). Each organ that performs its normal functions is composed of distinct sphingolipid hydrocarbons, suggesting that increased peaks of unique sphingolipid hydrocarbons^52^ imply a damaged organ or inflamed tissue, facilitating the diagnosis of human disease. Homeostasis among subsets of sphingolipids, such as ceramides, sphingosines, S1P, C1P, and SPC, is important for the maintenance, progression, and regulation of the immune response^7,118^. We analyzed reported sphingolipid-related human diseases from the PubMed Gene database in Table 1 and visualized the information with Fig. 2 to facilitate understanding of human diseases from the aspect of sphingolipid metabolism. The small counts of numbers in Fig. 2 among the diagrams imply that further extensive human research is required for the diagnosis of human disease. Therefore, we hope that future extensive studies on sphingolipid-mediated signaling in the immune system will result in an improvement in human health.
